# Cortical cytasters: a highly conserved developmental trait of Bilateria with similarities to Ctenophora

**DOI:** 10.1186/2041-9139-2-23

**Published:** 2011-12-01

**Authors:** Miguel Salinas-Saavedra, Alexander O Vargas

**Affiliations:** 1 Laboratory of Ontogeny and Phylogeny, Department of Biology, Faculty of Science, University of Chile. Las Palmeras, Ñuñoa, Casilla 653, Santiago, Chile

**Keywords:** cytasters, cytoplasmic asters, parthenogenesis, polyspermic fertilization, bilaterian, ctenophores, microtubule network, centrioles.

## Abstract

**Background:**

Cytasters (cytoplasmic asters) are centriole-based nucleation centers of microtubule polymerization that are observable in large numbers in the cortical cytoplasm of the egg and zygote of bilaterian organisms. In both protostome and deuterostome taxa, cytasters have been described to develop during oogenesis from vesicles of nuclear membrane that move to the cortical cytoplasm. They become associated with several cytoplasmic components, and participate in the reorganization of cortical cytoplasm after fertilization, patterning the antero-posterior and dorso-ventral body axes.

**Presentation of the hypothesis:**

The specific resemblances in the development of cytasters in both protostome and deuterostome taxa suggest that an independent evolutionary origin is unlikely. An assessment of published data confirms that cytasters are present in several protostome and deuterostome phyla, but are absent in the non-bilaterian phyla Cnidaria and Ctenophora. We hypothesize that cytasters evolved in the lineage leading to Bilateria and were already present in the most recent common ancestor shared by protostomes and deuterostomes. Thus, cytasters would be an ancient and highly conserved trait that is homologous across the different bilaterian phyla. The alternative possibility is homoplasy, that is cytasters have evolved independently in different lineages of Bilateria.

**Testing the hypothesis:**

So far, available published information shows that appropriate observations have been made in eight different bilaterian phyla. All of them present cytasters. This is consistent with the hypothesis of homology and conservation. However, there are several important groups for which there are no currently available data. The hypothesis of homology predicts that cytasters should be present in these groups. Increasing the taxonomic sample using modern techniques uniformly will test for evolutionary patterns supporting homology, homoplasy, or secondary loss of cytasters.

**Implications of the hypothesis:**

If cytasters are homologous and highly conserved across bilateria, their potential developmental and evolutionary relevance has been underestimated. The deep evolutionary origin of cytasters also becomes a legitimate topic of research. In Ctenophora, polyspermic fertilization occurs, with numerous sperm entering the egg. The centrosomes of sperm pronuclei associate with cytoplasmic components of the egg and reorganize the cortical cytoplasm, defining the oral-aboral axis. These resemblances lead us to suggest the possibility of a polyspermic ancestor in the lineage leading to Bilateria.

## Background

**Figure 1:**
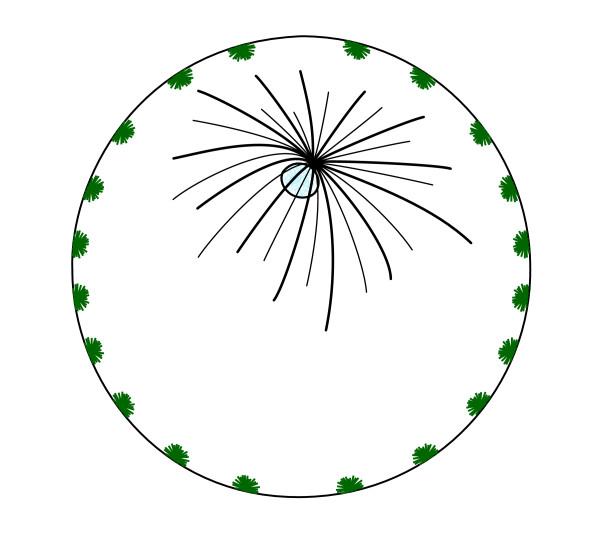
**Schematic representation of a section of the bilaterian egg**. There are two cytoskeletal domains of microtubules in the first interphase of the bilaterian zygote: one formed by cytasters (asters, painted green) placed in the cortex or surface of the egg (Ectoplasmic domain), and a second domain organized by the monoaster (black aster) of the sperm-derived centrosome, placed in the inner cytoplasm (Endoplasmic domain). Discrete foci schematically represent the centrioles (these can only be observed in actual embryos by means of special techniques, such as the injection of labeled tubulin or application of microtubule-polymerizing agents like taxol).

The zygote of Bilateria is known to have well-differentiated and independent cytoskeletal domains of microtubules that divide the cytoplasm in two regions, the ectoplasmic (cortical) and endoplasmic (inner) domains (Figure 1) [1-5]. The endoplasmic domain presents a single aster (monoaster, black aster in Figure 1), whose radially running microtubule fibers are nucleated from the centrosome [6]. Upon fertilization, the sperm centriole becomes the new endoplasmic centrosome (the maternal centrosome is no longer observable)^a^. The sperm-derived centrosome then reorganizes the endoplasmic domain: movements of inner cytoplasm polarize the egg, segregating the maternal components from yolk and transporting them towards the animal pole, where the centrosome is found [1,4,7-9]. The centrosome also forms the mitotic spindle, driving early cell divisions. In the ectoplasmic domain of the egg, developmental studies often describe the presence of numerous cytasters. These are microtubule-organizing centers that can be observed by means of techniques that induce microtubule stabilization and polymerization (such as the application of Taxol and Nocodazole) as foci surrounded by a star-like aggregation of microtubules, hence their name (cytoplasmic asters) [10-12]. Sometimes, agents for microtubule stabilization may produce star-like structures that are not true nucleating centers of microtubules, but are formed because short stabilized microtubules slide against each other, as revealed by ultrastructural and immunofluorescence analysis ([13]. For an example see reference [14]). This is decidedly not the case with the cortical cytasters observed in the egg of Bilateria. Like centrosomes, cortical cytasters contain centrioles that have been observed repeatedly using electron microscopy [11,12,15-17]. Cytasters, however, differ from centrosomes in that the latter consist of two orthogonally arranged centrioles that are surrounded by pericentriolar matrix; in contrast, cytasters are composed of one to several associated centrioles, without a specific arrangement [15-17]. Like centrosomes, the foci of cytasters are hard to observe *in vivo*. Without applying stabilizing agents like Taxol, the cortical microtubule cytoskeleton (ectoplasm) has the *in vivo *appearance of a homogeneous network. However, this 'homogeneous' cortical cytoskeleton is in fact formed by networks of interconnected cytasters [1,10-12,18-22]. In the case of the leech [1,20] and of the wasp *Nasonia vitripennis *[23], the discrete foci of cytasters are observable *in vivo *by microinjection of labeled tubulin, and in the egg cortex of *Drosophila*, foci are observable using immunofluorescence without Taxol [24,25].

Despite repeated descriptions of cytasters in animal eggs from different bilaterian phyla, there has been no formal review of their potential developmental and evolutionary relevance. Indeed, cortical cytasters appear to play a crucial role in early development: after fertilization, cortical cytoplasm undergoes reorganization, with movement of cytoplasmic components that are independent from the movements of endoplasmic reorganization ([7] and references therein). The movements of cortical cytoplasm are crucial to the patterning of the antero-posterior and dorso-ventral body axis ([4,9,26] and references therein). A well-known example is cortical rotation and grey crescent formation after fertilization in early amphibian development. The cytoskeleton is required for these cytoplasmic movements, and cytasters are presumed to play a crucial role [27-29]. Cytasters conceivably can act in cytoplasmic reorganization much like the centrosome does during cell division, positioning and transporting various cytoplasmic components [30-33].

## Presentation of the hypothesis

We propose that cytasters are an ancient and highly conserved trait of Bilateria that was already present in the most recent common ancestor of Protostomia and Deuterostomia (the "Urbilateria"). Thus, we propose homology and conservation of bilaterian cytasters, in contrast with the alternative possibility of homoplasy: that cytasters evolved independently in different bilaterian groups.

Detailed descriptions of cytasters in oogenesis and early development are published for several 'model system' Bilateria, particularly so sea urchins, amphibians, and holometabolous insects such as Diptera and Hymenoptera. The available information allows us to compare the extent of similarity in the formation, structural dynamics and function of cytasters observed in both deuterostome and protostome taxa. Specific similarities would suggest that an independent evolutionary origin of cytasters is unlikely, providing a first argument for the antiquity and homology of cytasters in Bilateria. In contrast, if the processes of cytaster formation were found to be essentially different for different phyla, this could be consistent with the possibility of independent origins (although it would not prove homoplasy *per se*). Different mechanisms of cytaster formation would suggest that cytasters could form easily under different biological conditions, supporting the argument for homoplasy.

## Developmental similarities in protostomes and deuterostomes

**Figure 2:**
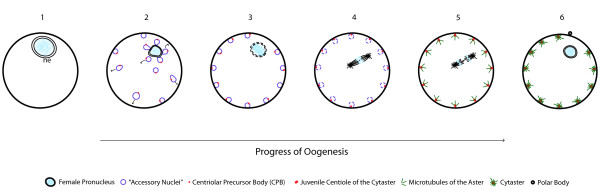
**The development of cytasters**. During oogenesis **(1 to 6)**, multiple accessory nuclei (purple) detach from the nuclear envelope (ne) and migrate towards the egg surface **(2)**. The nuclear envelope breaks down **(**dashed line, **3) **and the centriolar precursor bodies (CPB, red points), which are placed in these membranous elements **(3 and 4)**, develop into the centrioles (red circles) of the cytasters, which simultaneously nucleate microtubules of the asters **(**green lines, **5)**. At the end of oogenesis **(6)**, cytasters (green) have surrounded the egg cortex. Arabic numbers represent the sequence of events (not stages of meiosis). The polar body indicates the end of the first meiosis.

**Figure 3:**
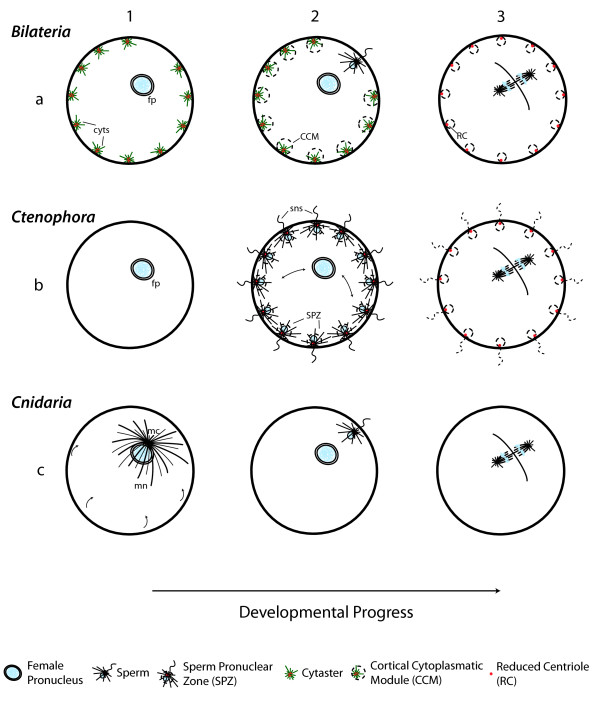
**Schematic illustration of early development in Cnidaria, Ctenophora, and Bilateria**. In bilaterian eggs **(1 to 3a)**, cytasters (cyts) are present in the oocyte (during and at the end of oogenesis; **1c**). When the egg is activated by fertilization **(1a)**, cytasters reorganize the ectoplasm around them forming the cortical cytoplasmic modules (CCM, dashed circle) and mitosis starts. Immediately following mitosis **(3a)**, cytasters begin to lose their distinct radial configurations, gradually reverting back to small astral areas that diminish considerably in size (dashed line), possibly becoming reduced centrioles (RC). Bilaterian cytasters have a similar role to that of sperm pronuclei in ctenophores. In Ctenophora **(1 to 3b)**, previous to the entry of sperm **(1b) **no cytoplasmic movements are observed. When supernumerary sperm (sns) enter the egg **(2b)**, cytoplasm reorganizes into several sperm pronuclear zones (SPZ, dashed circle) and the female pronucleus (fp) chooses one sperm (double arrow). In this moment cell division starts and supernumerary sperm asters diminish in size and become integrated to the microtubule network (dashed line; **3b**). In Cnidaria, normal development **(1 to 3c) **requires a microtubule network (mn) exclusively organized by the maternal centrosome (mc; **1c**), and after fertilization **(2c)**, by the sperm-derived centrosome. All maternal determinants necessary for normal development are transported towards the animal pole (arrows in **1c**), where the nucleus and centrosome are found. Fertilized eggs of cnidarians contain a residual microtubule network at the time of first mitosis **(3c)**.

In both protostome and deuterostome taxa, cytasters first become visible during oogenesis (Figure 2). Their centrioles have been observed to develop from Centriolar Precursor Bodies (CPBs) associated with the nuclear envelope, which act as 'seeds' for centrioles, as described for Hymenoptera [34], Echinodermata [15,35-39], and Mammalia ([40-43]). These 'seeds' probably correspond to centrin buds, accumulations of centrin proteins associated with the outer surface of the nuclear envelope, which have been well studied in mammalian culture cells ([44,45] and references therein). The first step in the ontogeny of cytasters is that numerous membranous elements containing CPB 'seeds' detach from the nuclear envelope and move from the cytoplasm to the oocyte cortex (2 in Figure 2). These membranous elements are called accessory nuclei in insects [34], annulate lamellae in sea-urchin [36] and sea-cucumber [38], multivesicular aggregates in mouse [40], and small vesicles in rabbit [42]. Initiation, assembly, and development of the centriole and aster of cytasters begin after the breakdown of the membrane of the nucleus (germinal vesicle) during meiosis (3 in Figure 2), when CPBs recruit maternal proteins from the oocyte cytoplasm and become centrioles (4 to in Figure 2). Diverse cytoplasmic components (mitochondria, endoplasmic reticulum, granular material, ribosomes, proteins, maternal mRNA, membranous elements, and others) then become associated with cytasters concomitant to the progress of meiosis [15,34,40]. New cytasters are also produced by centriole duplication of fully formed cytasters [24]. The formation of cytasters is completely independent in timing and place from centrosome duplication and nuclear division in the cell cycle [15,46]. After fertilization, and immediately following the first mitosis, cytasters begin to lose their distinct radial configuration and gradually revert back to small astral (star-like) areas that diminish considerably in size, eventually losing most of their characteristic astral features (3a in Figure 3). The centriole becomes no longer visible by electron microscopy, and is integrated within the ectoplasmic network of microtubules [15,37,47,48].

The fact that all the specific developmental processes above have been described in both protostome and deuterostome taxa suggests that cytasters did not originate independently in each lineage but are homologous, having already been present in their most recent common ancestor (the Urbilateria). The specific developmental pathways also suggest that cytasters are not easily formed under different biological processes and conditions. A clear prediction of the hypothesis of homology is that most basic groups of Bilateria should also conserve these specific similarities. In contrast, if taxa with cytasters are nested within groups that otherwise lack cytasters, this would provide evidence of homoplasy, with cytasters appearing independently in different lineages. The absence of cytasters, or their modification, in turn, can be proven to be a secondarily derived condition, if it occurs in taxa that are nested within groups that otherwise exhibit the presence and general developmental pattern of cytasters. By examining published evidence from non-bilaterian outgroups, such as Ctenophora and Cnidaria, we can also test whether cytasters originated exclusively in the lineage leading to Bilateria, or in the ancestors of a larger group of Eumetazoa. To this purpose, we reviewed published data reporting the presence or absence of cytasters in different animal species. Although some of the older descriptions are rudimentary, modern observations with plenty of structural detail are available for several taxa. The techniques used and structures observed are summarized in Additional file 1. This bibliographical review allows identification of the more important gaps in information and future directions of research that will allow further testing of the hypothesis of homology.

**Figure 4:**
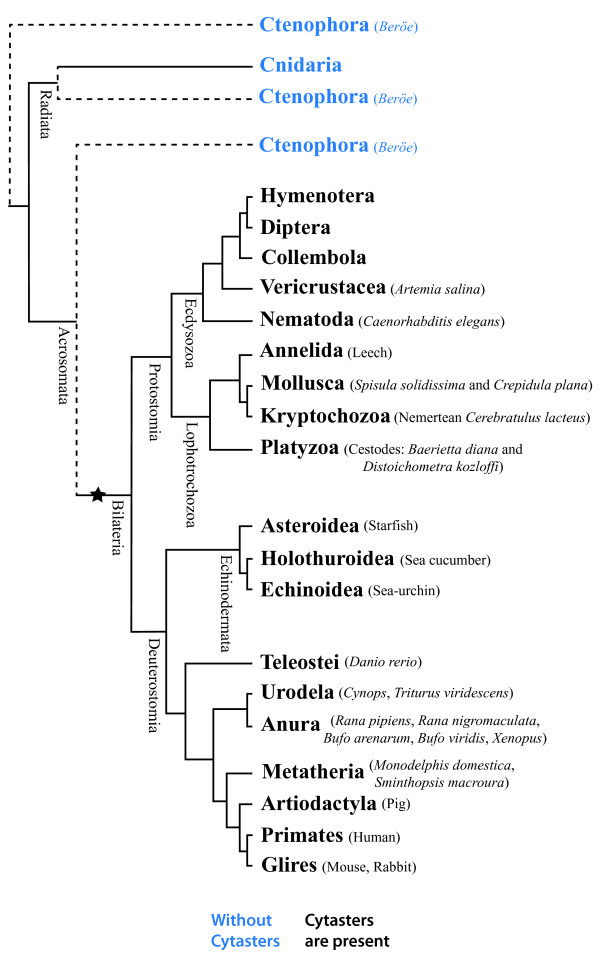
**Phylogenetic distribution of cytasters**. The tree shows different lineages where the absence or presence of cytasters has been determined by appropriate visualization techniques. Cytasters are absent in Cnidaria and Ctenophora (blue font), but have been described in a wide sample of both deuterostome and protostome lineages (dark font). The available data supports the hypothesis that cytasters evolved only in the lineage leading to Bilateria (asterisk in figure) and were already present in the most recent common ancestor of protostomes and deuterostomes. While the Ctenophora do not have egg cytasters, after polyspermic fertilization the centrosomes of the sperm carry out a similar role in re-organizing cortical cytoplasm and establishing the oral-aboral axis. The position of Ctenophora as a sister group to Bilateria is supported by morphological evidence but is controversial according to molecular evidence, with other possibilities indicated by dashed lines. This phylogeny was adapted from Stach (2008) [57] for chordate relationships, Regier *et al*. (2010) [104] for arthropod relationships, from Zrzavý *et al*. (1998) [100] for Ctenophora, and from Hejnol *et al*. (2009) [56], and Philippe *et al*. (2009) [97] for all other relationships.

The available information reveals a diversity of bilaterian phyla in which egg cytasters have been reported (see Figure 4), as summarized in Additional file 1. Both Deuterostomia and Protostomia are well represented by several lineages, including some that diverged very early within each group, as we describe below. The available information is also summarized in Additional file 1.

### Cytasters in protostomes

Within protostomes, it is well accepted that two main lineages exist, the Lophotrochozoa and the Ecdysozoa. The Lophotrochozoa includes well-studied phyla such as Mollusca and Annelida. In zygotes of the leech (an annelid) the cortical cytoplasm is populated by numerous interconnected cytasters that together constitute the whole cortex microtubule network. This ectoplasmic cytoskeletal domain is observable in the egg before fertilization, from the meiotic phase onwards, when no monoaster has yet been formed, which confirms its formation is independent of the monoaster [1]. The reorganization and translocation of the ectoplasmic cytoskeleton is linked to the dynamics of cytasters (see [20]). No studies have yet confirmed (or discarded) the initial formation of cytasters from nuclear vesicles (See Additional file 1). In the mollusk *Crepidula plana*, as in the aforementioned groups, cytasters are in the egg cortex, surrounded by cytoplasmic components, closely associated with vesicles (presumably of nuclear origin), and their formation is not related to the centrosome [49] (See Additional file 1). Another mollusk in which cytasters have been reported is *Spisula solidissima *[50]. These observations are important since the lineage leading to annelids and mollusks (the Lophotrochozoa) diverged from that leading to insects (the Ecdysozoa) towards the origins of Protostomia. Other Lophotrochozoa in which cytasters have been observed (although in less detail) are the Cestoda *Baerietta diana *and *Distoichometra kozloffi *[51], and the Nemertean *Cerebratulus lacteus *[52].

Within the Ecdysozoa, information about cytasters is available in great detail for holometabolous insects like Hymenoptera (as discussed above) and Diptera. They have also been described in Collembolla, indicating that cytasters were already present at the origin of the Hexapoda [17,23-25,53,54]. In other arthropods, cytasters have been described in the eggs of the Vericrustacean *Artemia salina *[5]. The description of cytasters in the nematode *Caenorhabditis elegans *[55] is important, because Nematodes diverged early from all other Ecdysozoan lineages [56]. We conclude that the available evidence is consistent with the homology and conservation of egg cytasters in Protostomes, according to a good sample of lineages spanning both Ecdysozoa and Lophotrochozoa.

### Cytasters in deuterostomes

The deuterostomes are split into two main groups, the Ambulacraria and the Chordata [56,57]. Within the Ambulacraria, cytasters are especially well-studied in sea urchins, as discussed above ([15,16,18,21,58,59] and references therein). In sea urchins, interconnected cytasters [21] take part in cytoplasmic rotation (cortical reorganization over 16°C) when the egg is fertilized [58]. Cytasters are also present in other echinoderms such as starfish [39], sea cucumber [38], and sand-dollar [35,60]. Within the chordates, available information is almost entirely restricted to amphibians and mammals. Otherwise, one of us has confirmed the presence of cytasters in the egg cortex of the teleost *Danio rerio*, the zebrafish (MS personal observation). In the amphibian *Cynops *(a newt), the cytasters are distributed around the whole unfertilized egg cortex, and form a coarse microtubule network in parallel array, except around the meiotic spindle at the animal pole (cytoplasm is restricted to the animal pole, because of the high yolk content at the vegetal pole; [48]). This parallel array correlates with the direction of cortical rotation. Cytoplasmic asters also have been described in the oogenesis of the salamander *Triturus viridescens *[61] and several anurans (see Additional file 1), related to cortical and germ plasm movements [11,12,27,62-65].

In mammals, as expected, the cytasters surround the cortex of the unfertilized egg [22,47,66-69] and are independent of the sperm derived-centrosome. Cytasters have been described in the oocytes of human [70], pig [43,71], rabbit [42], opossum [72] and marsupial rat [73]. Observations in marsupials are especially interesting, since this lineage diverged early within mammals. The Glires (mouse and rabbit) deserve special attention because they are derived: their cytasters do not contain mature centrioles. Rather, the nuclear vesicles (multivesicular aggregates) have been observed to contain CPBs surrounded by pericentriolar matrix [40-42]. The absence of a mature centriole has led to the description of these cytasters as acentriolar [19,74], but it must be kept in mind that CPB can also organize microtubule polymerization (even in absence of pericentriolar matrix [15]). Navara [75] reported that no cytasters are present in the cow. If this is not an artifact, and the cow is really lacking cytoskeletal organizers in the cortical cytoplasm, this would represent a secondary loss of cytasters in evolution: Phylogenetically, the cow is firmly nested within mammals that do have cytasters.

### Cytasters in absence of the centrosome: a common role throughout Bilateria

In the early development of Bilateria, the centriole of the sperm provides the centrosome, which drives the migration, encounter, and fusion of the sperm and egg pronuclei as well as the early cycles of cell division. However, in some cases where the sperm-derived centrosome is absent, these functions are carried out by cytasters. As we have described above, the Glires differ from other mammals because their cytasters do not have mature centrioles (see above). Glires are also derived in that the oocyte has no centriole [69], and there is no sperm-derived centrosome in the fertilized egg. In the absence of a sperm-derived centriole to form the centrosome, the only comparable organizers are cytasters, which take over its function in guiding the migration of the pronuclei towards the center of the egg [67]. Cytasters in Glires also organize and form the mitotic spindle, allowing the cell cycles of early mouse development [19,22,67-69,74,76] in the absence of a centrosome. Subsequently, at the blastocyst stage, in each cell a cytaster develops into a mature centriole and becomes a centrosome ([19,40,42] and references therein). This demonstrates that despite being described as acentriolar, the cytasters of Glires can be functional as cytoplasmic organizers and have the potential to become the centrosome. The functional takeover of centrosomal functions by cytasters has also been repeatedly observed in several bilaterian phyla in cases in which the sperm-derived centrosome may be absent yet development proceeds. This is the case for many Hexapoda that present natural parthenogenesis [17,23-25,53,54] and is also true for experimentally induced parthenogenesis in eggs of the Vericrustacean *Artemia salina *[5]. In sea urchin and sand dollar, when eggs are artificially activated (without fertilization), the cytasters form a structure resembling a mitotic spindle (bipolar but without asters; [59]) and development proceeds parthenogenetically [35,37]. Cytasters also take over the function of early cell divisions when parthenogenesis is artificially induced in the pig and rabbit [42,43]. In the salamander *Triturus*, in experiments on androgenic development, eggs are fertilized in the absence of the sperm-derived centrosome, so cytasters must organize the early cell divisions [61]. In all the cases mentioned above, cortical reorganization and cell divisions proceed normally, in the absence of a sperm-derived centrosome. This demonstrates that cytasters can organize and move cytoplasmic components much like a centrosome, which supports the strong inference that cytasters are crucial for the reorganization of cortical cytoplasm and axis establishment. A potential for replacing centrosomal functions in early development, and a capacity to eventually become centrosomes, is another specific trait of cytasters that is ubiquitous across distantly related bilaterian phyla (see Additional file 1).

### Development without cytasters: Cnidaria and Ctenophora

A review of published evidence from basic groups of Eumetazoa supports the notion that cytasters are an exclusively bilaterian trait. Outside of Bilateria, in Cnidaria and Ctenophora, no cytasters are formed during the process of oogenesis, as confirmed by immunofluorescence and microtubule polymerization techniques [14,77] (Figure 4). In cnidarians, animal-vegetal (a-v) polarity of the oocyte is generated during oogenesis and is present in the oocyte before fertilization, with the animal pole at the site of emission of the polar bodies [77,78]. Maternal determinants that specify the germ line (for example, *Nvvas1*, and *Nvnos2 *RNA [79]) and oocyte polarity (*CheFz1 *RNA [77]) are transported throughout the entire cytoplasm involving microtubules [77,79], and not in the ectoplasm alone as in Bilateria [3,8,80-86]. There is no independent ectoplasmic network of microtubules, nor did we find any description suggesting the differentiation of cortical cytoplasm in cnidarians. In this case, the polarization of the egg is directed by the centrosome associated with the meiotic spindle. Transportation and reorganization of maternal determinants in cnidarians begins during oogenesis and is completed after fertilization, mediated by a single microtubule network that is required for their transport (1c in Figure 3) [77]. This microtubule network has no obvious polarity, other than the position at the animal pole of the maternal centrosome of the meiotic spindle during oogenesis. At the moment of fertilization, the maternal centrosome is absent and the sperm-derived centrosome localizes at the animal pole (2c in Figure 3) [77]. At the end of each meiotic and mitotic cycle, when the centrosome ceases its activity, the entire microtubule network is depolymerized [77], in contrast with bilaterian eggs, in which the cortical network remains. The position of the centrosome of the meiotic/mitotic spindle (both before and after fertilization) is associated with the direction of transportation of maternal determinants during ooplasmic segregation. Under normal conditions, all maternal determinants necessary for normal development are transported towards the animal pole (1c in Figure 3) [78], where the nucleus and centrosome of the meiotic/mitotic spindle are found [77]. When centrifuged, the centrosome can be moved to a position that is offset from the nucleus. In this case, maternal determinants are now transported towards the new position of the centrosome (rather than the nucleus). The presence of a single microtubule network nucleated by a single centrosome in the egg [77] confirms that cnidarians do not have an independent ectoplasmic network of microtubules comparable to that formed in bilaterians and ctenophores. Thus, the process of redistribution of maternal determinants in cnidarian eggs involves only the meiotic/mitotic spindle in cytoplasmic reorganization, rather than multiple cytasters. In this sense, we may compare cnidarian development to the endoplasmic domain of Bilateria, which is also reorganized upon fertilization by the microtubule network associated with the sperm-derived centrosome. In contrast, in both Ctenophora and Bilateria, reorganization of cortical cytoplasm is independent of the centrosome.

### Cortical reorganization without cytasters: The role of polyspermy in Ctenophora

Cytasters are absent in the eggs of ctenophores (Figure 4) as confirmed by immunofluorescence and microtubule polymerization techniques [14]. However, cortical cytoplasmic movements after fertilization occur that closely resemble those of bilaterian ectoplasm (1 to 3b in Figure 3). The ctenophore *Beroe ovata *has physiological polyspermic fertilization [87,88] taking place immediately before or during formation of the first polar body (1 to 2b in Figure 3) [89]. After several spermatozoa enter the egg, cytoplasmic components become associated with the supernumerary sperm cells, each forming a spherical zone called Sperm Pronuclear Zone (SPZ; 2b in Figure 3), that consists of cortical granules, mitochondria, endoplasmic reticulum, and other cytoplasmic components, including the nuclear envelope of the sperm pronucleus. Each SPZ is organized by the centriole associated with each male pronucleus [89]. The formation of this new cortical cytoplasmic configuration is carried out by microtubule-mediated waves and is of utmost importance in establishing the oral-aboral axis [14], which takes place after the fusion of pronuclei and first cleavage cycle [14,89]. In the polyspermic Ctenophora, it is the female pronucleus of *Beroe *that migrates to join a stationary male pronucleus, choosing one (2b in Figure 3). At this site, the zygote nucleus forms, first mitosis occurs and the first cleavage furrow starts [14,89]. As development and cell division advances, the nuclear envelopes of the supernumerary male pronucleus break down, their DNA is degraded, and the giant asters nucleated by their associated centrosomes shrink and are no longer visible, presumably becoming integrated with the microtubule network (3b in Figure 3) [14]. The centrioles of zygote centrosomes retain their mature form.

## Testing the hypothesis

**Figure 5:**
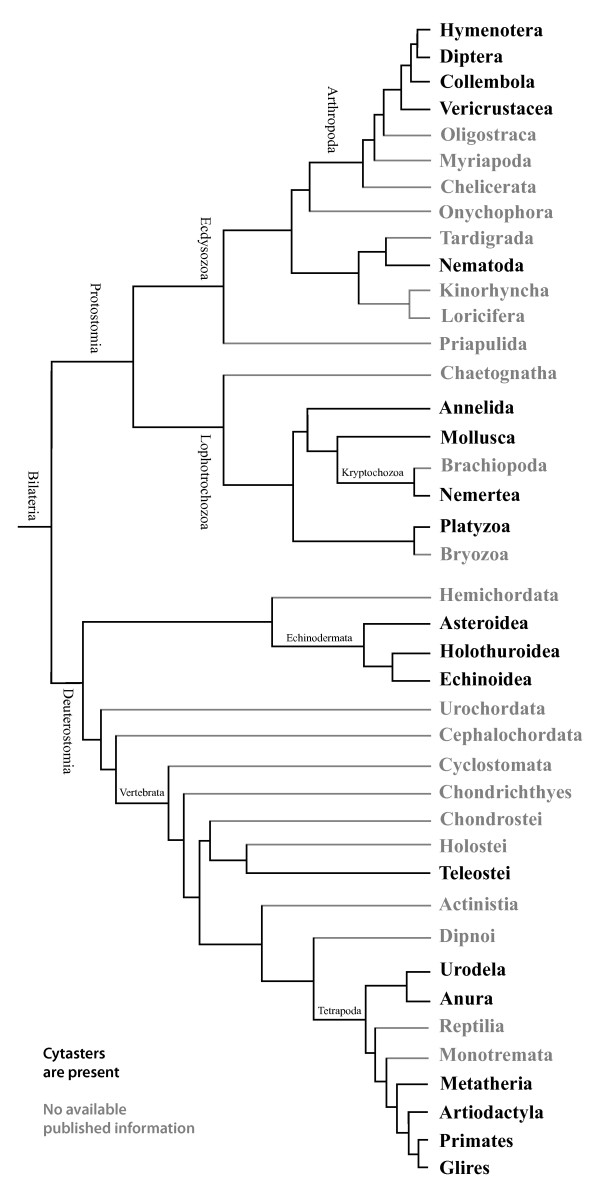
**Bilaterian groups expected to present cytasters**. Cytasters have been described in a wide sample of both deuterostome and protostome phyla (dark branches). Grey branches are used for phyla for which we found no available published information. More detailed studies are necessary to clarify the presence or absence of cytasters in these missing phyla, using modern techniques uniformly applied across different taxa. This phylogeny was adapted from Stach (2008) [57] for chordate relationships, Regier *et al*. (2010) [104] for arthropod relationships, and from Hejnol *et al*. (2009) [56], and Philippe *et al*. (2009) [97] for all other relationships.

The specific developmental similarities of cytasters across Bilateria provide compelling evidence of homology, a hypothesis that is also supported by the available data on phylogenetic distribution of the presence of cytasters. Published information on the presence of cytasters covers a very good taxonomic sample: 20 orders, representing 15 classes and 8 phyla (see Figure 4, Additional file 1). For perspective, we can consider how genomic structure and molecular-developmental aspects are typically known for poor taxonomic samples of only a few model species. Good taxon sampling is crucial to any hypotheses of homology, because it allows testing for evolutionary patterns such as conservation, homoplasy, and secondary loss. We found no evolutionary pattern that would support the independent origin of cytasters in different bilaterian phyla. We found a single (possible) case of absence of cytasters: The cow. Further confirmation of this case is important, since this would be a clear case of a secondary loss of cytasters in evolution. Also within mammals, the Glires are interesting since they show how small differences may evolve in a specific lineage (lack of sperm-derived centrosome, cytasters with immature centrioles), while at the same time conserving the general pattern of formation and function of cytasters. In all, published available data is overwhelmingly consistent with homology and conservation of cytasters across bilateria. Homology directly implies that cytasters should be observable in several groups for which there is currently no available published information, that are represented in Figure 5 by grey branches (Dark branches represent groups in which the presence of cytasters has already been documented). Within the Deuterostomes, data are missing for the phyla Hemichordata, Urochodata and Cephalochordata. Within Vertebrata, data are missing for several basic groups of fishes: Cyclostomata, Chondrichthyes, Chondrostei, Holostei, Actinistia, and Dipnoi. Within Tetrapoda, no data are available for Monotremata and Reptilia (including birds). In Protostomes, data are missing for several important phyla: Chaetognatha, Bryozoa, Brachiopoda, Kinorhyncha, Loricifera, Priapulida, Onychophora, Tardigrada, Chelicerata, Myriapoda, and Oligostraca (Pancrustaceans). In some groups, like Platyzoa [51] and Kryptochozoa [52], the available information about cytasters was described during the early twentieth century by observations of fixed and stained eggs made under the light microscope. Updated descriptions are desirable to confirm that these groups really present cytasters. We found no available information about the presence or absence of cytasters in the eggs of Placozoa and Porifera. The documented absence of cytasters in the other non-bilaterian phyla Cnidaria and Ctenophora suggests that cytasters should also be absent in Placozoa and Porifera. If so, this would support the notion that cytasters only evolved in the lineage leading to Bilateria. For all the phyla mentioned above, new studies on the presence or absence of cytasters can benefit from uniform application of modern techniques, ensuring sound comparison of data across phyla.

## Implications of the hypothesis

### Cortical cytoplasmic modules are an ancient and highly conserved Bilaterian trait

Based on the observations in several different bilaterian animals, cytasters are much more than star-like centers of microtubule polymerization. They have a specific ontogeny, developing from precursor bodies (possibly centrin buds) found in fragments of nuclear membrane (often described as accessory nuclei or vesicles) that move to the egg cortex and become associated to several cytoplasmic components, conforming complex cortical modules (Figure 2, Additional file 1). Thus, to go beyond the notion contained in the descriptive term cytaster, we wish to forward the notion and acronym of *Cortical Cytoplasmatic Modules *(CCM) for the entire assemblage of centriole (or, as in mouse and rabbit, centriolar precursor) associated with the membranous element, aster and associated cytoplasmic components: mitochondria, endoplasmic reticulum, granular material, ribosomes, proteins, maternal mRNA, membranous elements, and others.

### Are CCMs inherited in cytoplasm?

The development of CCMs brings up interesting questions about their inheritance. An intriguing possibility suggested by Kallenbach and Mazia [37] is that the oocyte cytasters upon disappearing may produce themselves the CPBs (seeds) that become allocated to the nuclear surface. Thus, cytaster reduction may be an important source of seeds that become centrioles of new CCMs during oogenesis^b^. In Bilateria, during the process of cell division, the nuclear membrane (from which vesicles of CCM's are formed) disintegrates into fragments, which thereafter allocate to the cytoplasm of both resulting cells [30,32,33,90]. Thus, it is easy to conceive how CPBs could be inherited through cytoplasm containing these fragments. CPBs may always be present in the nuclear membrane, but only become cytasters during the process of oogenesis. If so, artificial induction of cytaster formation is conceivable. This seems to be the case in cultured mammalian cells, where formation of cytasters similar to that in oogenesis is induced upon the experimental ablation of the centrosome [44] and when cells are arrested in S-phase [45]. In these cell cultures, the centrioles of cytasters develop from seeds in vesicles of the nuclear membrane that move towards the cortical cytoplasm. These seeds in the nuclear membrane have been shown to be centrin buds containing alpha/gamma-tubulin and centrin 2. Like centrioles, these seeds are capable of self-replication [45], and are probably the same as the CPB seeds observed in oogenesis. Further research is required to establish if new centrin buds can only be formed by replication, or can also be assembled from isolated centrin proteins [44,91]. If new CPBs only form by replication, they can only be inherited through cytoplasm.

### A polyspermic ancestor?

The early development of Bilateria can be compared to other Eumetazoan outgroups in order to make inferences about the origin of CCMs. In this regard, the capacity of the sperm-derived centrosome to re-organize the egg cytoplasm (as observed in Cnidaria and the endoplasm of Bilateria) is linked in the case of the polyspermic Ctenophora to the establishment of a well-differentiated ectoplasmic domain, which is required for the establishment of the oral-aboral axis. Compelling resemblances exist between the CCMs of bilaterian eggs and the aforementioned SPZ of Ctenophora. The SPZ and CCM both possess a microtubule-nucleating centriole (and aster) initially associated with the nuclear membrane (sperm pronucleus of SPZ, and vesicles of nuclear membrane or accessory nuclei in CCM), which becomes surrounded by associated cytoplasmic components of the egg. Both are required for the early developmental processes of ectoplasmic reorganization and axes establishment, and both have a similar developmental fate, reducing the aster and centrioles, which become incorporated in the microtubule network (see above; compare illustration 2-3b and 2-3a in Figure 3). As discussed above, in the absence of the sperm-derived centrosome, CCMs are capable of taking over its role in the migration, encounter, and fusion of the sperm and egg pronuclei [92]. In the ctenophore *Beroe ovata*, when the unfertilized egg is artificially activated (SPZs are absent) the female pronucleus migrates randomly in the cytoplasm throughout the egg [14,93]. When the egg of *Beroe *is fertilized, SPZs drive the migration, encounter, and fusion of pronuclei [93]. As discussed above, when bilaterian oocytes are artificially activated (no sperm enter the egg), they undergo the normal process of segregation and redistribution of cytoplasm, without reactivation of meiosis ([30,33] M. Salinas-Saavedra, personal observation in zebrafish and sea urchin) and development often continues parthenogenetically [17,24,35,48,53,54,73,94,95]. In contrast, cytoplasmic reorganization and cell division is disrupted in artificially activated eggs of ctenophores, demonstrating that SPZs are required for the same processes carried out by CCMs in Bilateria.

The remarkable similarities to ctenophore SPZs listed above suggest that the development and function of CCMs are derived from early developmental processes similar to those in the polyspermic Ctenophora. More specifically, we propose that the origin of CCMs involved the acquisition by the female germ line of the capacity to produce numerous centriole-based modules of ectoplasm, a process that previously was required for the exogenous contribution of supernumerary sperm. Because CPBs can be inherited through cytoplasm, it is possible that the female germline acquired numerous CPBs directly from the reduction of sperm centrioles. An interesting point to observe in future studies is the distribution of germline determinants (like *vasa *and *nanos*) during the development of polyspermic Ctenophores. According to our hypothesis, we expect a cortical localization of these markers associated with the microtubules of *SPZs*, similar to the distribution observed in bilaterian eggs, associated to microtubules of the egg cortex.

The phylogenetic relationships of Ctenophora to other metazoa are currently controversial [56,96-102]. However, Ctenophora has often been suggested to be a sister group to Bilateria [96,99-102], especially on the basis of the morphological evidence. If so, this would support the notion that polyspermy could have been present in the most recent common ancestor of Ctenophora and Bilateria, and the lack of a differentiated cortical cytoplasm, as in cnidarians, may represent a primitive condition for the egg (with a single microtubule network).

The complex egg of Bilateria and its reorganization are crucial in the selective distribution of cytoplasmic domains during cleavage, leading to body axis patterning. Despite the well-acknowledged importance of egg cytoplasmic domains and their movements [1,4], their evolutionary origin is seldom discussed and thus seems largely mysterious. In this sense, a new explicative framework emerges when we consider the possibility that these cytoplasmic movements may derive from a complex, polyspermic fertilization, like that of Ctenophora, leading to the differentiation of an ectoplasmic domain and the cytoplasmic movements of body axis patterning. More detailed research in Ctenophora and Cnidaria is bound to be informative about the early evolutionary history of cytoplasmic reorganization.

## List of abbreviations

CCM: cortical cytoplasmatic module; CPB: centriolar precursor body; cyts: cytasters; fp: female pronucleus; mc: maternal centrosome; mn: microtubule network; RC: reduced centriole; sns: supernumerary sperm; SPZ: sperm pronuclear zone.

## Competing interests

The authors declare that they have no competing interests.

## Authors' contributions

MS and AOV wrote the article and were responsible for comparative and evolutionary interpretation of the data. MS made the figures and additional file material. Both authors read and approved the final version of the manuscript.

## Endnotes

^a^Previously, during the formation of the egg, the endoplasm is organized by the germinal vesicle-derived centrosome. In the literature, authors discussing the fate of the oocyte-derived centrosome state that it is missing at the end of meiosis [30,53].

^b^This notion is supported by the experimental injection of centrioles (isolated from adult tissues) into the egg of *Xenopus*. These centrioles become reduced and cease to be observable, but upon fertilization, an increased number of CCMs shows up at the site of injection ([103] and references therein). This suggests the injected centrioles provided an increased number of centriole precursor bodies at the injection site.

## Supplementary Material

Additional file 1 **Characteristics of cytasters in different taxa**. Tables that summarize the available information on cytasters in Protostomia and Deuterostomia, indicating observed structural aspects and the techniques used. The bold font represents taxa in which the specific developmental pathway of cytaster formation is well documented. Click here for file
