# Supplementary material for: Cortical cytasters: a highly conserved developmental trait of Bilateria with similarities to Ctenophora
Source: EvoDevo. 2011 Dec 1;2:23. doi: 10.1186/2041-9139-2-23 (PMC3248832; doi:10.1186/2041-9139-2-23)
Supplement: Additional file 1 — Characteristics of cytasters in different taxa. Tables that summarize the available information on cytasters in Protostomia and Deuterostomia, indicating observed structural aspects and the techniques used. The bold font represents taxa in which the specific developmental pathway of cytaster formation is well documented. [file 2041-9139-2-23-S1.PDF]

A) Protostomes

		Cortical asters (Cytasters) in egg	Centrioles were observed by MET	Cytasters are associated to cytoplasmic components	Origin of cytasters associated to fragments of nuclear vesicle	Not derived from centrosome	Cortical cytoplasmic components are reorganized	Capacity to become a centrosome	Technique	Reference
**Hexapoda**	**Insects, Collembola**	Present	Present	Present	Present	Present	Present	Present (in natural parthenogenesis)	Immunofluorescence and MET	17, 24, 25, 53, 54.
Vericrustacea	*Artemia Salina*	Present	Data Not Available	Data Not Available	Data Not Available	Present	Present	Present (in natural parthenogenesis)	Light microscopy observations of fixed embryos and oocytes	5
Nematoda	*Caenorhabditis elegans*	Present	Data Not Available	Data Not Available	Data Not Available	Present	Data Not Available, but suggested by authors	Data Not Available	Immunofluorescence in taxol-treated embryos	55
Annelida	Leech	Present	Data Not Available	Present	Data Not Available	Present	Present	Data Not Available	Immunofluorescence in live and fixed oocytes	1, 6, 20
Mollusca	*Spisula solidissima*	Present	Data Not Available	Present	Data Not Available	Present	Unclear	Data Not Available	Light microscopy observations in taxol-treated oocytes	50
	Crepidula plana	Present	Data Not Available, but are inferred.	Present	Present	Present	Present	Present, in mitosis without cellular divisions (amitosis)	Light microscopy observations in normal and hypertonic conditions	49
Kryptochozoa	*Cerebratulus lacteus*	Present	Data Not Available	Data Not Available	Present	Present	Data Not Available	Data Not Available	Light microscopy observations of whole eggs and eggs fragments	52
Platyzoa	*Baerietta diana, Distoichometra kozloffi*	Present	Data Not Available	Data Not Available	Data Not Available	Present	Data Not Available	Data Not Available	Light microscopy observations in fixed and stained eggs	51

B) Deuterostomes

		Cortical asters (Cytasters) in egg	Centrioles were observed by MET	Cytasters are associated to cytoplasmic components	Origin of cytasters associated to fragments of nuclear vesicle	Not derived from centrosome	Cortical cytoplasmic components are reorganized	Capacity to become a centrosome	Technique	Reference
**Echinodermata**	**Sea-urchin**	Present	Present	Present	Present	Present	Present	Present (in artificial parthenogenesis)	Immunofluorescence, light microscopy, and MET in normal, hypertonic and low temperature conditions, and treatment of eggs with several drugs	15, 18, 21, 37, 16, 58, 59.
	Sand-dollar	Present	Present	Data Not Available	Present	Present	Present	Present (in artificial parthenogenesis)	Detailed embryological descriptions using light microscopy of the oocyte and embryo under normal, hypertonic and low temperature conditions and treatment of eggs with several drugs	35, 60
	Starfish	Present	Data Not Available, but suggested for authors	Data Not Available	Present	Present	Data Not Available	Data Not Available	Immunofluorescence	39
	Sea cucumber	Present	Present	Present	Present	Unclear	Unclear	Data Not Available	Light microscopy, MET, and treatment of eggs with several drugs	38
Amphibians	*Cynops*	Present	Data Not Available	Data Not Available	Data Not Available	Present	Unclear	Data Not Available	Immunofluorescence, Immunohistochemistry, light microscopy and treatment of eggs with several drugs	48
	Triturus viridescens	Present	Data Not Available	Data Not Available	Data Not Available	Present	Data Not Available	Present (in androgenic development)	Observations by light microscopy in induced androgenic development	61
	Rana pipiens	Present	Data Not Available	Present	Data Not Available	Present	Data Not Available	Data Not Available	Light microscopy observations at low temperature	62
	Rana nigromaculata	Present	Data Not Available	Present	Data Not Available	Present	Present	Data Not Available	Whole egg and cytological sections observations by light microscopy in centrifuged embryos and activated egg with frog's blood	63
	Bufo arenarum	Present	Present	Present	Data Not Available	Present	Present	Data Not Available	Injections of centriole preparation and cytological examination by light microscopy	65
	Bufo viridis	Present	Data Not Available	Data Not Available	Data Not Available	Present	Data Not Available	Data Not Available	Cytological examination by light microscopy	64
	Xenopus	Present	Present	Present	Data Not Available	Present	Present	Data Not Available	MET, light microscopy observations in normal eggs and eggs treated with several drugs. Injections of mature centriole preparation	11, 12, 28, 46, 103
Fishes	Zebrafish	Present	Data Not Available	Data Not Available	Data Not Available	Present	Data Not Available	Data Not Available	Immunofluorescence against tyrosinated alfa-tubulin	M. Salinas-Saavedra observation
**Mammals**	**Mouse**	Present (astral foci from centriolar precursors, see text)	Present (Precursor)	Present	Present	Present	Present	Present	Embryological description by immunofluorescence, light microscopy, and MET in normal, treated with several drugs, hypertonic and low temperature conditions	19, 22, 40, 41, 47, 66-69, 76
	Human	Present	Data Not Available	Data Not Available	Data Not Available	Present	Data Not Available	Data Not Available	Immunofluorescence of parthenogenetic eggs treated with taxol	70
	**Pig**	Present	Data Not Available	Present	Present	Present	Present	Present (in artificial parthenogenesis)	Immunofluorescence and Immunohistochemistry of normal and parthenogenetic embryos treated with several drugs. Electric activation	43, 71
	Rabbit	Present (astral foci from centriolar precursors, see text)	Present (Precursor)	Present	Associated to a smooth membrane vesicles (nuclear or Golgi)	Present	Present	Present (in artificial parthenogenesis)	Embryologic and cytological observations by light microscopy and MET	42
	Monodelphis domestica	Present	Data Not Available	Data Not Available	Data Not Available	Present	Data Not Available	Data Not Available	Immunocytochemical observation during oogenesis; Treated and non-treated with taxol	72
	Sminthopsis macroura	Present	Data Not Available	Data Not Available	Data Not Available	Present	Data Not Available	Data Not Available	Immunocytochemical observation during oogenesis; Treated and non-treated with taxol	73
